# Incidental finding of APC deletion in a child: double trouble or double chance? – a case report

**DOI:** 10.1186/s13052-021-00969-x

**Published:** 2021-02-15

**Authors:** Erica Rosina, Berardo Rinaldi, Rosamaria Silipigni, Luca Bergamaschi, Giovanna Gattuso, Stefano Signoroni, Silvana Guerneri, Alessandra Carnevali, Paola Giovanna Marchisio, Donatella Milani

**Affiliations:** 1 Fondazione IRCCS Ca’ Granda Ospedale Maggiore Policlinico, Pediatric Highly Intensive Care Unit, Via della Commenda, 9, 20122 Milan, Italy; 2 Fondazione IRCCS Ca’ Granda Ospedale Maggiore Policlinico, Laboratory of Medical Genetics, Milan, Italy; 3 Pediatric Oncology Unit, Fondazione IRCCS Istituto Nazionale dei Tumori, Milan, Italy; 4 Department of Radiology, Fondazione IRCCS Ca’ Granda Ospedale Maggiore Policlinico, Milan, Italy; 5 Department of Pathophysiology and Transplantation, University of Milan, Milan, Italy

**Keywords:** 22q11.2 deletion syndrome, *APC*, Hepatoblastoma, DNA microarray, Incidental finding, Case report

## Abstract

**Background:**

22q11.2 deletion syndrome is one of the most common genomic disorders, characterized by the variable presence of facial dysmorphisms, congenital cardiac defects, velopharyngeal insufficiency/cleft palate, thymic hypoplasia/aplasia, immunodeficiency, parathyroid hypoplasia, developmental delay, learning disabilities, psychiatric disorders, renal, ocular, and skeletal malformations, hearing loss and laryngeal abnormalities. Chromosomal microarray (CMA) hybridization is one of the most performed diagnostic tests but as a genome wide analysis, it can point out relevant incidental copy number variations.

**Case presentation:**

We report the case of a 2-year-old boy that came to our attention for mild psychomotor delay, poor growth, and minor facial anomalies. Considering a diagnosis of 22q11.2 deletion syndrome, we performed CMA that not only confirmed our diagnosis, but also pointed out an additional de novo 5q21.3q22.2 microdeletion, encompassing *APC* gene. As a result of the genetic testing we enrolled the patient in a tailored surveillance protocol that enabled the early detection of a hepatoblastoma. The child underwent surgical and chemotherapic treatments with complete cancer eradication.

**Conclusions:**

The concurrent finding of an expected result and an additional deletion of *APC* gene represents an example of a relevant issue about the health and ethical management of secondary findings revealed by genome-wide tests. Furthermore, this report highlights the need to develop dedicated surveillance guidelines for children with *APC*-related polyposis and raise the question whether to suspect and screen for *APC*-related conditions in cases of sporadic hepatoblastomas.

## Background

Nowadays, chromosomal microarray (CMA) represents the first-tier genetic test for patients presenting with intellectual disability (ID) and/or multiple congenital anomalies of unknown origins, detecting DNA copy-number variants (CNVs), i.e. microduplications or microdeletions [1].

Among the pathogenic CNVs, 22q11.2 deletion syndrome (22q11.2DS) (OMIM #192430) is one of the most common microdeletion disorders: its incidence is relatively high and it is estimated in 1:4000 newborns, possibly underestimated due to clinical mis/under-recognition. The main clinical findings include facial dysmorphisms, congenital cardiac defects, velopharyngeal insufficiency with or without cleft palate, thymic hypoplasia/aplasia, immunodeficiency and/or autoimmune disorders, parathyroid hypoplasia, developmental delay (commonly borderline intellectual function), learning disabilities, psychiatric disorders, renal, ocular, and skeletal malformations, hearing loss and laryngeal abnormalities [2]. The most common heterozygous microdeletion, leading to DiGeorge/Velocardiofacial syndromes, encompasses ∼3 Mb, while 8–10% of individuals show a ∼1.5 Mb nested deletion, resulting in similar but overall milder phenotype [3]. In 90% of cases the deletion occurs sporadically, resulting from a de novo heterozygous deletion, while in the remaining 10% of individuals it is inherited, in an autosomal dominant pattern, from a parent occasionally showing mild features of the condition [2].

22q11.2DS can be diagnosed using FISH Test (Fluorescence In Situ Hybridization) with specific probes mapping the region, or with whole-genome methodologies, such as CMA. The first one can be beneficial on a strong clinical suspect but it could misdiagnose patients with atypical nested deletions [2]. CMA might be more straightforward for patients with subtle clinical presentation and/or for less experienced health professionals; however, as a genome wide analysis, it can unveil incidental microdeletions/microduplications, possibly relevant for the patient, that have to be properly communicated and managed.

Here we present a child with clinical suspect of 22q11.2DS and whose diagnostic process led to an incidental genetic finding with important consequences on the clinical management and outcome.

## Case presentation

The male proband is the second child to non-consanguineous healthy parents of South-American ancestry. The family history is not contributive. Pregnancy was uneventful and the baby was born at term with a weight of 2600 g (10th centile), length of 48 cm (25th centile), occipitofrontal circumference (OFC) of 32 cm (10th centile), Apgar score of 9/10. An umbilical hernia was evident. His growth was normal until 6 months old, when he showed a progressive slowdown. His parents reported frequent infections of upper respiratory tract, treated with antibiotic therapy.

During a hospitalization for a gastroenteritis at the age of 19 months, he underwent a neurological examination that showed a delayed psychomotor development (he could say only one word and he did not walk alone), hypotonia, excessive sleepiness and poor socialization. Magnetic resonance imaging (MRI) of the brain and brainstem revealed minor dysmorphic aspect of ventricular system, asymmetrical and slightly verticalized hippocampi and slightly reduced anterior-posterior diameter of pons. Cardiac and endocrinological evaluation were normal. Routine blood tests showed a microcytic anemia caused by iron deficiency, mild hypoalbuminemia and hypocalcemia. When the child was 21 months old, during a second hospital recovery for herpetic stomatitis, an otorhinolaryngologist evaluation was performed for nocturnal snoring and tonsillectomy was suggested.

**Fig. 1:**
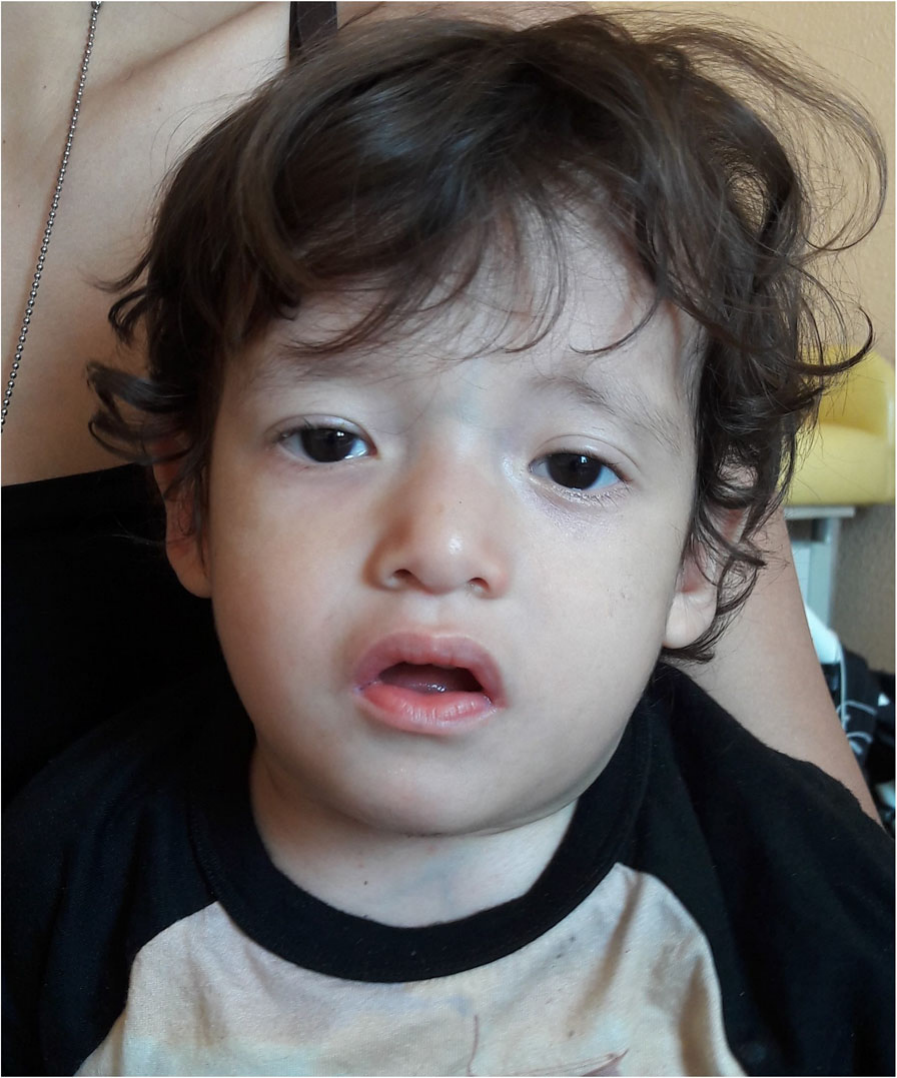
Photograph of the patient at 2 years old. Minor facial anomalies reminiscent of 22q11.2DS are notable, including bitemporal constriction, low-set ears, sparse eyebrows, short palpebral fissures, bilateral epicanthic folds, depressed nasal bridge, tubular nose and mild hypoplastic alae nasi

For the mild psychomotor delay, poor growth and brain anomalies he was referred to the genetic consultant. On his first genetic evaluation, his weight was 9.5 kg (<3rd percentile) and his head circumference was 46 cm (<3rd percentile). Minor facial anomalies suggestive of 22q11.2DS were observed, including bitemporal constriction, low-set ears, sparse eyebrows, short palpebral fissures, bilateral epicanthic folds, depressed nasal bridge, tubular nose and mild hypoplastic alae nasi (Fig. 1).

Because of the medical history and the facial appearance, a CMA analysis was performed on the patient and his parents. The CMA analysis was performed using a 60-mer oligonucleotide probes technology (SurePrint G3 Human CGH 8x60K, Agilent Technologies, Santa Clara, CA, USA) on DNA extracted from peripheral blood. Labeling, purification and hybridization of DNA samples were carried out according to manufacturer’s protocol (Agilent Oligonucleotide Array-Based CGH for Genomic DNA Analysis, version 7.5). Raw data were generated using Agilent Feature Extraction and analyzed by CytoGenomics 4.0.3.12 using ADM-2 algorithm (Agilent Technologies, Santa Clara, CA). To improve the accuracy of the results the Diploid Peak Centralization algorithm was applied.

The aberration filter was set to detect a minimum number of 3 consecutive probes/region and the minimum absolute average Log Ratio (MAALR) was ± 0,25. A second analysis was run with a MAALR of ±0,15 and with a minimum number of 3 probes/region to detect low level mosaicism.

Copy number variations weren’t reported if they coincided with published DNA variants listed in the Database of Genomic Variants (http://projects.tcag.ca/variation/). Genomic coordinates are in accord to the 37 build (March 2009) of the Human Genome Reference consortium (GRch37/hg19).

**Fig. 2:**
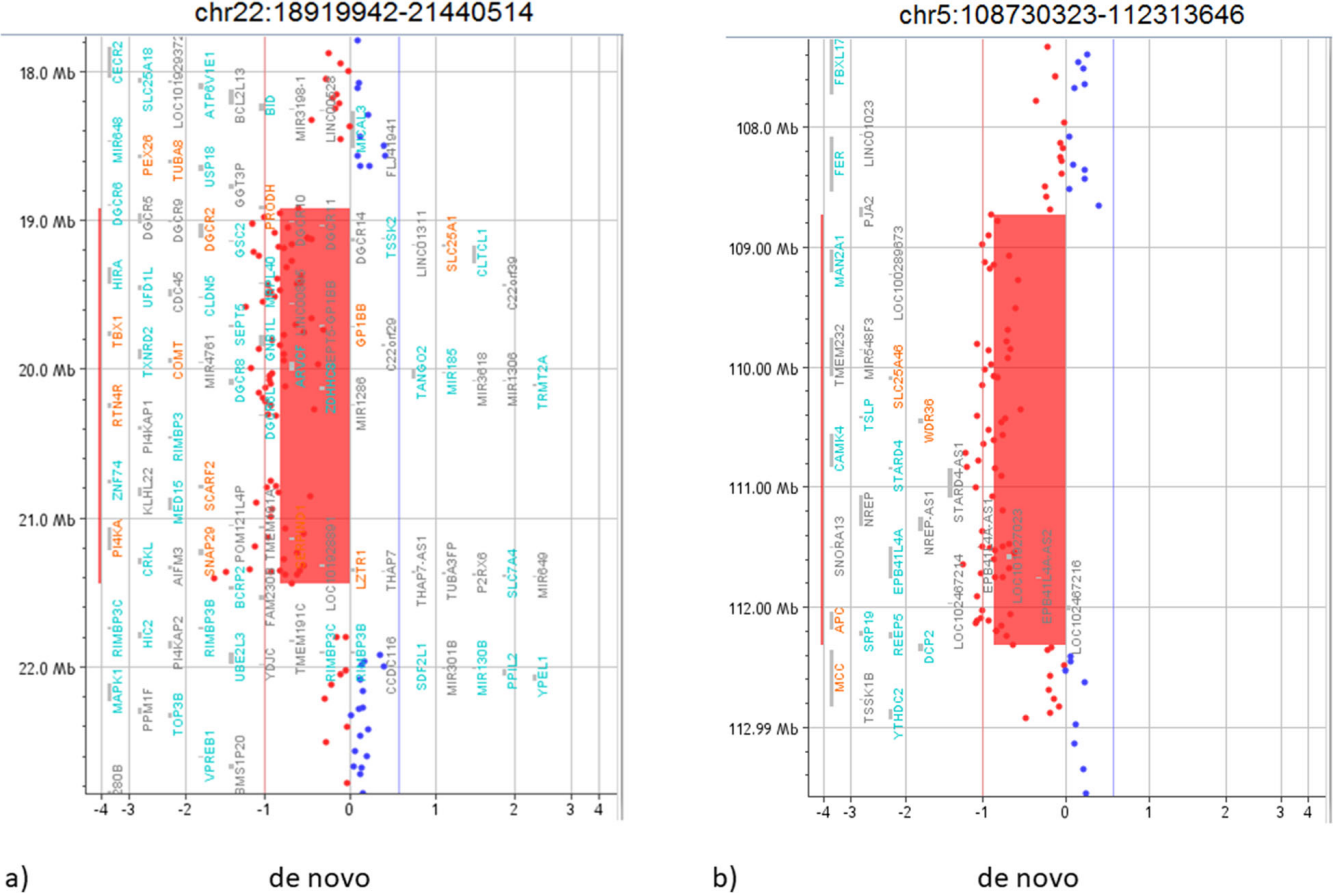
Pathogenic CNVs of the patient. **a** It is shown part of array-CGH results of the patient, focusing on the 2.5 Mb deletion on the long arm of chromosome 22 at band q11.21 that involve 43 OMIM genes. **b** It is represented the 3.6 Mb deletion on the long arm of chromosome 5 at band q21.3q22.2 that involve 12 OMIM genes, including *APC*. The breakpoints are reported according to the 37 build (March 2009) of the Human Genome Reference consortium (GRch37/hg19)

The analysis detected four rearrangements (Table 1): the small sizes of the two microduplications, their genic content and the parental origins were suggestive for a likely benign role. One de novo rearrangement was a typical 22q11.21 microdeletion associated to the 22q11.2 deletion syndrome (Fig. 2a). Furthermore, a de novo 5q21.3q22.2 microdeletion was identified: this 3.6 Mb deletion involves 12 OMIM genes, including *APC*, delineating a condition of *APC*-associated polyposis (Fig. 2b).

**Table 1:** Summary of patient’s CNVs detected by chromosomal microarray

Chromosome region	Copy number variation	Size	Breakpoints^**a**^	OMIM disease-causing genes involved	Inheritance	Role
2q34	Duplication	432 kb	210,021,463–210,453,149	/	Father	Likely benign
5q21.3q22.2	Deletion	3.6 Mb	108,730,323–112,313,646	*SLC25A4*, *WDR36*, *APC*	De novo	Pathogenic
7q21.12	Duplication	307 kb	87,811,283–88,118,091	*ADAM22*	Mother	Likely benign
22q11.21	Deletion	2.5 Mb	18,919,942–21,440,514	*PRODH, SLC25A1, CDC45L, GP1BB, TBX1, TXNRD2, COMT, TANGO2, RTN4R, SCARF2, PI4KA, HCF2*	De novo	Pathogenic

^a^The breakpoints are reported according to the 37 build (March 2009) of the Human Genome Reference consortium (GRch37/hg19)

**Fig. 3:**
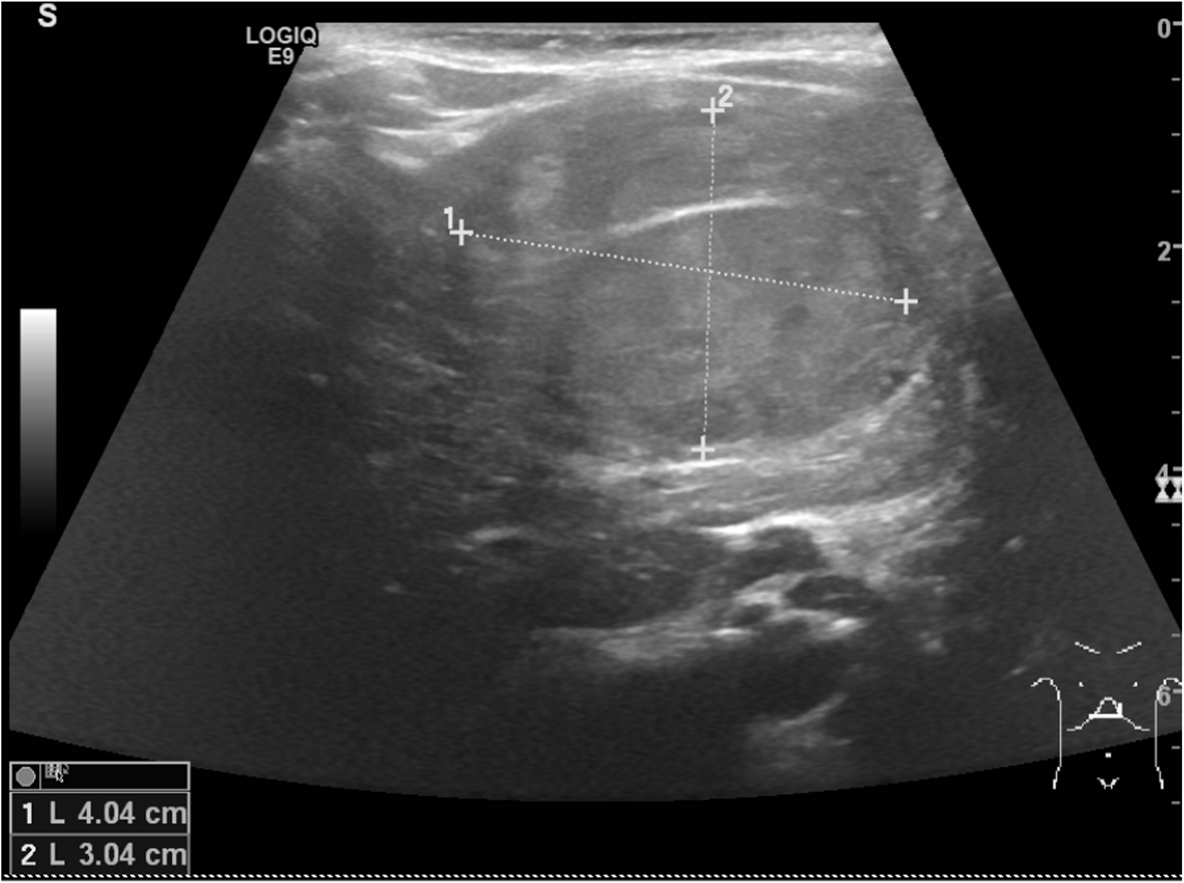
Abdomen ultrasound scan of the patient’s liver showing the hepatoblastoma. The figure shows a scan of the abdominal ultrasonography of the patient: it revealed a solid mass of 4 × 3 cm in the left lobe of the liver, with well-defined edges, ovular and bilobate shape

As a result of the genetic test the proband was enrolled for a tailored follow-up. At the first evaluation, an abnormally high alpha-fetoprotein (αFP) level was detected (266.4 μg/L, normal range < 7 μg/L) and the abdomen ultrasound showed a solid mass of 4 × 3 cm in the left lobe of the liver, with well-defined edges, ovular and bilobate shape (Fig. 3). For a more accurate evaluation of the lesion, a magnetic resonance (MRI) of the abdomen was performed and it confirmed the presence of a liver mass, in II-III-IV segment. It appeared irregularly hyperintense in T2-wheighted images and hypointense in T1, with multinodular structure and a delimiting pseudocapsule, hypointense in both T2 and T1 sequences. The endovenous paramagnetic contrast agent showed an irregular enhancement that increased in venous phase, with persisting hyperdense strie in the late-phase. This lesion, according to the irregular pattern of enhancement, structure and vessel relation, was compatible with a diagnosis of hepatoblastoma (HB).

The patient then was referred to the Pediatric Oncology Unit of Istituto Nazionale dei Tumori. Clinical examination was unremarkable. Blood investigations confirmed an increased αFP level (97.5 μg/L); beta human chorionic gonadotropin hormone (βhCG) value was normal. Chest x-ray was normal, without evidence of any thoracic lesion. Considering these assessments, a core-needle biopsy of the mass was performed and a diagnosis of fetal epithelial hepatoblastoma was made.

Following this diagnosis, the child was treated according to the current guidelines for very low risk hepatoblastoma patients. First of all, a left hepatic lobectomy was performed (resection of segments II-III), without any post-surgical complications. Histological examination confirmed the diagnosis and the radicality of the surgery (fetal epithelial hepatoblastoma; mitotic index > 2/10 high power fields (HPF); tumor-free resection margins). After that, considering the histological evidences, two cycles of adjuvant chemotherapy with cisplatin (100 mg/m^2^ in a continuous intravenous 48-h infusion) were administered with a 21 days interval, starting 1 month after surgery. The αFP values gradually decrease during therapy and finally normalized 2 months after the end of chemotherapy. The treatment was not complicated by haematological or other toxicities except for an episode of upper respiratory tract infection, after the first cycle of chemotherapy, probably favoured by the predisposition determined by 22q11.2DS.

Eighteen months after the end of treatment, the child had no evidence of tumor recurrence. He continued with a periodical specific oncological follow-up including clinical evaluation, radiological assessment (abdominal ultrasonography) and serum αFP dosage; besides, he was referred to specialists of inherited gastrointestinal cancer syndromes for the appropriate FAP surveillance. He also continued the genetic follow-up with hematologic tests, eye examination and thyroid ultrasound. At the latest evaluation, thyroid functional parameters were altered: he was referred to the endocrinologist for appropriate diagnostic framework and treatment.

## Discussion and conclusions

The array-CGH analysis confirmed the clinical suspect of 22q11.2DS and led to an incidental finding of a cancer predisposition syndrome due to the deletion of the *APC* gene.

Germline haploinsufficiency of the *APC* gene cause familial adenomatous polyposis (FAP), a highly penetrant condition characterized by development of various rectal and colonic adenomas and early-onset colorectal cancer [4]. The majority of disease-causing alterations of the *APC* gene are loss-of-function single nucleotide variants [5]; large cytogenetic deletions, containing *APC* gene, have been rarely reported and it is estimated that they account for 2–3% of patients [6, 7]. The *APC* gene germline mutations and deletions are not only involved in colorectal tumorigenesis, but also in other various premalignant and malignant lesions: duodenal, jejunal and gastric polyps, papillary thyroid carcinomas, desmoid tumors and embryonal tumors, such as hepatoblastoma (HB) and medulloblastoma. Whole *APC* gene deletions seem to cause mainly classical-FAP phenotype, with thousands of adenomatous colonic polyps and high frequency of extraintestinal FAP related manifestations such as upper gastrointestinal polyps, congenital hypertrophy of the retinal pigment epithelium (CHRPE) and desmoid tumors [6, 8, 9].

The 22q11.2 microdeletion identified in our proband could explain the clinical features of delayed psychomotor development, hypotonia, poor socialization, frequent episodes of infectious diseases, abnormal findings at electroencephalogram and encephalic MRI, hypocalcemia and hypothyroidism and characteristic facial dysmorphisms. Although 22q11.2DS has not been clearly associated with an increased risk of malignancy, few cases of pediatric cancers have been reported among these patients [10–12]: in particular Scattone et al. [10] and McDonald-McGinn et al. [12] described respectively one and two patients with 22q11.2DS and hepatoblastoma. The distinctive immunodeficiency, predisposing to higher rate of infectious diseases also from carcinogenic viruses and to impaired tumor surveillance, together with the heterozygous deletions of some specific genes involved in detoxification of carcinogenic substances (such as catechol-O-methyltransferase gene) are possible concurrent causes in the complex process of carcinogenesis [11].

In this scenario, 22q11.2DS might have contributed to the HB development along with *APC* deletion that remain the prevalent predisposing factor for our patient. In fact, the increased risk of developing HB in patients carrying a constitutional mutation or deletion of *APC* is estimated to be really low, ranging from 0.3 to 1.6% [13], but it is approximately 750–7500 times higher the prevalence of general population (0.0001%) [14]. The median age at diagnosis of HB in FAP children is similar to that of sporadic ones, predominantly between 6 months and 3 years of age [15]. Since the prognosis of HB is strictly dependent on early detection and, consequently, on the complete resection, screening test in patients with germline haploinsufficiency of *APC* is mandatory. Beyond increasing survival, it could allow less-intensive therapy and less organ toxicity. To date, there is not a standard agreement regarding time and method of surveillance. Given the estimated risk, similar to other genetic predisposing conditions (such as Beckwith-Wiedemann syndrome), several studies suggest αFP level monitoring in conjunction with hepatic ultrasound at least every 3 months [16], until 7 years of age [13–17].

This and previous reports also bring into question whether to suspect and screen for *APC*-related conditions in cases of sporadic hepatoblastomas. HB accounts for about 1% of all pediatric tumors and represents the most common liver cancer in childhood: if our case had not been tested before, his HB would be classified as “sporadic” one, as he had no suggestive family history or other manifestations of FAP. Aberrant activation of *Wnt*-signaling pathway occurs in the vast majority of HBs through somatic mutations at beta-catenin gene (*CTNNB1*, OMIM *116806), particularly point mutations or in-frame deletions involving exon 3 [16–18]; APC protein is a key negative regulator of *Wnt*-signaling pathway. The reported rate of germline *APC* mutations in apparently sporadic HBs is highly variable: Sumazin et al. [18] found approximately 1,1% of sporadic HBs with an *APC* germline mutation, while Aretz et al. [15] and Young et al. [19] reported an incidence of 10–20%, therefore suggesting routine *APC* testing of all patients with HB. Since somatic *CTNNB1* and germline *APC* mutations have been shown to occur in a mutually exclusive manner in hepatoblastomas and in all tumor types studied so far [18–20], the identification of a somatic activating *CTNNB1* mutation in an HB patient directly reduces the risk of carrying a germline *APC* mutation [20]. Based on these data, a two-step approach would be preferable: testing for somatic mutations of *CTNNB1*, trough immunochemistry and/or genetic analyses on tumor blocks, should be performed firstly; then all *CTNNB1*-negative patient should undergo screening for germline *APC* mutation/deletion with a combination of sequencing methods and MLPA (multiplex ligation-dependent probe amplification). This must be a first-tier analysis instead in cases with extra-liver FAP manifestations or a suggestive family history.

Like *APC*, many other genes and correlated syndromes have a well-known increased risk to cancer in pediatric age; at least 8–10% of them reveal a germline mutation in cancer predisposition genes known at this days, with *APC* as the second most frequent mutated gene in this type of patients [21]. Detailed guidelines for genetic analyses in apparently sporadic childhood cancers are missing: testing all pediatric cancer patient for germline cancer predisposition genes could be a waste in resources and most importantly could lead to a notable number of unknown significant variants with the consequent massive family impact that these type of “uncertain diagnoses” bring with them. Instead, as the first step, somatic tumor screening can not only provide information about distinct molecular risk subtype and help clinical management, but it can also aid in distinguishing sporadic tumors from those related to a genetic predisposition syndrome.

Our case is a perfect example of genetic “double diagnosis” made with a genomic testing like CMA: if we would confirm the clinical suspect of 22q11.2DS using FISH technique, we would miss the *APC* deletion with important repercussion on the patient’s care. Nowadays, genome-wide tests, such as CMA and whole exome sequencing (WES), have not only considerably increased the diagnostic yield but the rate of unexpected and uncertain findings as well. Talking about CMA, an incidental finding could be defined as unexpected CNVs not directly related to the patient’s clinical indication that has however some medical implications, conferring susceptibility to cancer, neurodegenerative adult-onset pathologies or revealing a carrier status. These incidental findings are also relevant for the repercussions on other family members, often leading to critical testing in healthy-appearing individuals and raising emblematic ethical dilemmas such as testing siblings in childhood [22]. While, for exome and genome sequencing, whether and which incidental variants must be reported is an ongoing discussion topic [23], the contribution of CNVs is under-explored especially considering that it is currently used as the first diagnostic tool. Some large studies have investigated the proportion of incidental CNVs and their genetic counselling implications [24–27]. In particular, based on a literature meta-analysis by Talukdar et al., the incidence of CNVs involving cancer susceptibility genes (CSGs) in individuals that underwent CMA is 0.6% [17]. According this UK consensus group, microdeletions encompassing *APC* are categorised as “recognised deletion/duplication syndrome involving a cancer susceptibility gene with a demonstrable elevated lifetime risk of cancer evident from the literature, for which surveillance is recommended”, for which laboratory report is mandatory [17]. In the Italian setting, specific recommendations for this topic are lacking; however we strongly believe that, in pre-test counselling, the medical geneticist must inform, with an appropriate consent form, the patient or his parents about the possible incidental findings and they can freely decide whether to know. However if the patient’s willpower of not knowing is more acceptable for adult-onset diseases with no therapy or preventive care, finding an incidental CNVs that could lead to therapeutic/prevention measures, especially for children, open a strong ethical problem for clinicians [27]. For our case, not reporting the *APC* deletions would have been morally difficult and worrying for the patient’s health; and this made us realise how much international guidelines for incidental CNVs report are needed.

In conclusion, this report presents a 22q11.2DS child with an additional deletion in *APC* discovered with CMA. Involving a cancer predisposing condition, this incidental finding let us disclose it to the family, leading to an early diagnosis of hepatoblastoma and to its complete eradication. This case highlights some emerging health management and ethical issues about current genome-wide tests and incidental findings, even more notable being these analyses available also for non-genetic healthcare professionals. In this scenario, a tight collaboration among the different healthcare professionals involved in clinical management appears essential.

## Data Availability

Data sharing is not applicable to this article as no datasets were generated or analysed during the current study.

## Abbreviations

22q11.2DS: 22q11.2 deletion syndrome
αFP: Alpha-fetoprotein
βhCG: Beta human chorionic gonadotropine hormone
CMA: Chromosomal microarray
CNVs: Copy-number variants
CSGs: Cancer susceptibility genes
FAP: Familial adenomatous polyposis
FISH: Fluorescence In Situ Hybridization
HPF: High power fields
HB: Hepatoblastoma
ID: Intellectual disability
MAALR: Minimum absolute average Log Ratio
MRI: Magnetic resonance imaging
OFC: Occipitofrontal circumference
WES: Whole exome sequencing
