# Correction to: Incidental finding of APC deletion in a child: double trouble or double chance? – a case report

**DOI:** 10.1186/s13052-021-01135-z

**Published:** 2021-09-14

**Authors:** Erica Rosina, Berardo Rinaldi, Rosamaria Silipigni, Luca Bergamaschi, Giovanna Gattuso, Stefano Signoroni, Silvana Guerneri, Alessandra Carnevali, Paola Giovanna Marchisio, Donatella Milani

**Affiliations:** 1 Pediatric Highly Intensive Care Unit, Fondazione IRCCS Ca’ Granda Ospedale Maggiore Policlinico, Via della Commenda, 9, 20122 Milan, Italy; 2 Laboratory of Medical Genetics, Fondazione IRCCS Ca’ Granda Ospedale Maggiore Policlinico, Milan, Italy; 3 Unit of Hereditary Digestive Tract Tumors, Fondazione IRCCS Istituto Nazionale dei Tumori, Milan, Italy; 4 Department of Radiology, Fondazione IRCCS Ca’ Granda Ospedale Maggiore Policlinico, Milan, Italy; 5 Department of Pathophysiology and Transplantation, University of Milan, Milan, Italy

**Correction to: Italian Journal of Pediatrics 47, 31 (2021)**

**https://doi.org/10.1186/s13052-021-00969-x**

The original [1] article contains an incorrect affiliation for co-author, Stefano Signoroni.

The correct affiliation can be viewed in this Correction article, and should be considered instead.
